# Cell-based therapies for retinal diseases: a review of clinical trials and direct to consumer “cell therapy” clinics

**DOI:** 10.1186/s13287-021-02546-9

**Published:** 2021-10-11

**Authors:** John W. Hinkle, Raziyeh Mahmoudzadeh, Ajay E. Kuriyan

**Affiliations:** Wills Eye Hospital, Mid Atlantic Retina, Thomas Jefferson University, Philadelphia, PA USA

**Keywords:** Age-related macular degeneration, Cell therapy clinics, Human embryonic stem cells, Human umbilical tissue-derived cells, Induced pluripotent stem cells, Retinal pigment epithelium, Stargardt’s macular dystrophy

## Abstract

**Background:**

The retinal pigment epithelium (RPE) is implicated in the pathophysiology of many retinal degenerative diseases. This cell layer is also an ideal target for cell-based therapies. Several early phase clinical trials evaluating cell therapy approaches for diseases involving the RPE, such as age-related macular degeneration and Stargardt's macular dystrophy have been published. However, there have also been numerous reports of complications from unproven “cell therapy” treatments marketed by “cell therapy” clinics. This review aims to outline the particular approaches in the different published clinical trials for cell-based therapies for retinal diseases. Additionally, the controversies surrounding experimental treatments offered outside of legitimate studies are presented.

**Main body:**

Cell-based therapies can be applied to disorders that involve the RPE via a variety of techniques. A defining characteristic of any cell therapy treatment is the cell source used: human embryonic stem cells, induced pluripotent stem cells, and human umbilical tissue-derived cells have all been studied in published trials. In addition to the cell source, various trials have evaluated particular immunosuppression regiments, surgical approaches, and outcome measures. Data from early phase studies investigating cell-based therapies in non-neovascular age-related macular degeneration (70 patients, five trials), neovascular age-related macular degeneration (12 patients, four trials), and Stargardt’s macular dystrophy (23 patients, three trials) have demonstrated safety related to the cell therapies, though evidence of significant efficacy has not been reported. This is in contrast to the multiple reports of serious complications and permanent vision loss in patients treated at “cell therapy” clinics. These interventions are marketed directly to patients, funded by the patient, lack Food and Drug Administration approval, and lack significant oversight.

**Conclusion:**

Currently, there are no proven effective cell-based treatments for retinal diseases, although several trials have investigated potential therapies. These studies reported favorable safety profiles with multiple surgical approaches, with cells derived from multiple sources, and with utilized different immunosuppressive regiments. However, data demonstrating the efficacy and long-term safety are still pending. Nevertheless, “cell therapy” clinics continue to conduct direct-to consumer marketing for non-FDA-approved treatments with potentially blinding complications.

## Background

Cell-based therapies offer potential treatments for retinal degenerative diseases (RDD), including age-related macular degeneration (AMD), Stargardt's macular dystrophy (SMD), and retinitis pigmentosa (RP) [1–7]. These conditions affect patients at all stages of life. Acquired RDDs like AMD often manifest in older ages, while inherited degenerative diseases like SMD and RP manifest earlier. There is no Food and Drug Administration (FDA)-approved cell-based therapy to prevent the progression of a RDD, but emerging approaches hold promise for patients across all age ranges [8]. Phase I and II studies are investigating various cell sources, cell preparations, surgical methods, and immunosuppressive regimens in multiple diseases. However, these studies continue to evaluate safety primarily. Despite the lack of FDA-approved treatments, “cell therapy’ clinics offer unproven cell-based treatments to patients have become more widespread, and several reports have described severe complications from intraocular injections of “stem cells” [9]. The purpose of this review is to describe the data and issues related to cell-based therapy for RDDs in published clinical trials and to highlight the potential risks that “cell therapy” clinics pose to patients.

### Cell-based therapy sources

SMD and AMD, both the neovascular (NV-AMD) and non-neovascular (NNV-AMD) forms, have been targeted for treatment with cell-based therapies in clinical trials. The retinal pigment epithelium (RPE) is implicated in both conditions, and the RPE’s function does not depend on nerve synapses. These characteristics, along with the eye’s immune privilege, make the RPE a prime target for stem cell regeneration [10]. Various cell types have been used as the source of therapeutic cells, including human embryonic stem cells (hESCs), induced pluripotent stem cells (iPSCs), and human umbilical tissue-derived cells (hUTCs).

The induction of hESCs into retinal progenitor cells [11–13] has evolved since it was first proved feasible, and improved extracellular signaling techniques have made this process more efficient [14]. In recent cell-based therapy trials, hESCs remain the most prevalent source from which RPE cells are derived. However, the potential of these cells to differentiate into a wide variety of mature cells and to continue replicating indefinitely makes safeguarding these therapies important. Human trials continue to focus on confirming that the transplanted cells do not form teratomas, do not migrate into other organs, and do not have other unintended adverse effects [15]. While the ability of hESCs-derived RPE cells to be stored and readily available for any patient is an advantage, as these cells are not derived from the patient, immunosuppression therapy is necessary to prevent rejection [2].

iPSCs can also be induced to form RPE cells. In contrast to hESCs, iPSCs can be harvested from a previously differentiated cell source, such as skin. This characteristic reduces some of the ethical controversy surrounding hESCs, and these cells may be autologous rather than allogeneic. Because of this latter characteristic, iPSCs may decrease the risk of rejection and eliminate the need for systemic immunosuppression associated [2]. However, these cells would not be immediately available for treatment, as cells from a specific patient need to be collected, induced into iPSCs, and then induced to differentiate into RPE cells.

hUTDs are derived from extraembryonic mesoderm and can be used to treat neurologic disease without induced cell differentiation, making cell preparation technically simpler compared to hESCs and iPSCs [16]. These cell types do expand, but they cannot grow indefinitely in culture and do not spontaneously differentiate into other cells types like stem cells. Their function is likely secondary to secreted factors that support RPE and photoreceptor function (paracrine effect) [5, 16–18]. These cells can be readily available and stored for immediate use and may not require immunosuppression to prevent rejection.

Other cell therapy sources, including bone-marrow-derived stem cells and adipose-derived stem cells, are currently being investigated as well [19, 20].

### Cell-based therapy delivery

Regardless of origin, therapeutic cells can be delivered via several different techniques. Subretinal delivery can be accomplished with dissociated cells in suspension either through the retina after pars plana vitrectomy (PPV) or through a transscleral approach without vitrectomy [5, 21]. Alternatively, derived RPE cells can be cultured into a monolayer ex vivo, recreating the native structure. With this approach, the cells have apical and basal polarization maintained by tight junctions prior to implantation [3, 4]. These advantages are countered by a more complex surgical procedure and specific implantation technique associated with preformed monolayers. Adverse events that have been reported related to the surgical procedure include retinal tears and perforations and retinal detachments [3–7, 15, 22–24]. Intravitreal injection of cell therapies is also currently being investigated [25].

In addition to the risks inherent to the surgical procedure, there are specific safety concerns related to the use of these cells. One category of risk arises from the necessary ability of the cells to replicate. This can lead to excessive target tissue growth, replication in an unintended location, or teratoma formation. A second category of risk relates to an inflammatory response evoked by the use of allogeneic cells. The immune response inside the eye can range from vitritis, to membrane formation, to rejection of the transplanted tissue. However, in the absence of inflammation, cell atrophy alone [26] or progressive loss of function can indicate graft rejection [27]. In order to combat these immune reactions, systemic or local immunosuppression has been employed in all trials with cells derived from allogeneic sources, though some have tried to match donor and recipient HLA haplotypes. Although exact procedures vary from trial to trial, transplanted tissue itself undergoes multiple rounds of testing to confirm RPE purity, exclude residual pluripotency, and detect pathogens or other contaminants [15].

### Cell-based therapy trial endpoints

One major challenge in cell therapy trials is assessing the effectiveness of the transplants. To date, all clinical trials have been early phase studies of safety underpowered to detect functional changes. Nevertheless, each has attempted to assess the impact of the implanted cells. The worse-seeing eye has been uniformly selected, allowing the fellow eye to serve as an imperfect control. RPE cells are pigmented and are therefore identifiable on ophthalmoscopy. However, pigment can also be seen in macrophages scavenging dying RPE cells, and preclinical studies have demonstrated that only immunohistochemistry can reliably detect viable transplanted RPE [16, 28]. Therefore, in addition to reporting new areas of pigmentation, studies have relied on multimodal imaging. Optical coherence tomography (OCT) can demonstrate structural changes in the subretinal space as well as in photoreceptor outer segments. Fundus autofluorescence relies on lipofuscin, a product of normal retinal function that is absent in areas of atrophy, but is difficult to interpret after transplantation. Multifocal and full field electroretinogram (ERG) have been employed to assess retinal function. Visual field testing and microperimetry can be used to map the scotoma created by atrophy. Finally, visual acuity and reading speed can be tested, but some studies have focused on patients who have a very poor baseline vision, making these endpoints harder to utilize. Further development of visual function testing in low vision patients may be needed to assess efficacy of these treatments in end-stage disease patients.

### Published retinal cell-based therapy trials

#### Non-neovascular AMD (NNV-AMD)

NNV-AMD does not currently have any effective therapies, motivating many early phase trials to investigate cell-based therapies in this disease. Data have been reported for 72 patients who have undergone treatment in phase I and phase II studies, with follow-up for as long as 36 months (Table 1).

**Table 1:** Non-neovascular age-related macular degeneration trials

Trial	Disease	Sample size	VA inclusion criteria	Cell derivation and preparation	Transplantation approach	Immuno-suppression	VA outcomes	Adverse events (AE)
Palucorcel Phase 1/2a NCT01226628	NNV-AMD	35	Phase 1: ≤ 20/200 Phase 2a: ≤ 20/80	hUTC suspension	Suprachoroidal subretinal delivery	None	> 10 letter gain in 10/29 > 15 letter gain in 7/29	Retinal detachments in 6/35 Retinal perforations in 13/35 Subretinal delivery aborted due to retinal perforations during the surgical procedure in 2/35 AE related to surgery in 25/33 AE related to surgical delivery system in 19/33 AE related to cell suspension in 5/33
Palucorcel Phase 2b NCT02659098	NNV-AMD	21	20/80 to 20/800	hUTC suspension	Suprachoroidal subretinal delivery	None	Mean VA change: − 5.7 letters (vs. − 3.7 in control group) > 15 letter gain in 0/21	No serious AEs, no RDs, no perforations, no significant changes in IOP > 15 letters lost in 3/21
CPCB-RPE1 Phase 1/2a NCT02590692	NNV-AMD	5	≤ 20/200	hESC-RPE polarized monolayer	PPV and subretinal delivery	Tacrolimus	> 15 letter gain in 1/5 Stable vision in 4/5	Subretinal hemorrhage requiring bevacizumab treatment in 1/5 Implantation aborted due to subretinal debris 1/5
Advanced Cell Technology Phase 1/2a NCT01344993	NNV-AMD/ SMD	9 NN-AMD (3 dose escalation cohorts)	≤ 20/200	hESC-RPE suspension	PPV and subretinal delivery	Tacrolimus and MMF	> 15 letter gain in 4/9 10–14 letter gain in 2/9 Stable vision in 3/9	Preretinal pigmentation in 2/9
South Korean Phase 1/2a NCT01674829	NNV-AMD/ SMD	2 NN-AMD	≤ 20/320	hESC-RPE suspension	PPV and subretinal delivery	Tacrolimus and MMF	Improved VA in 1/2	Discontinuation of immunosuppression due to side effects in 1/2 Choroidal neovascular membrane in 1/2

*h-ESC* human embryonic stem cells; *HLA* human leukocyte antigen; *iPSCs* induced pluripotent stem cells; *MMF* mycophenolate mofetil; *NV-AMD* neovascular age-related macular degeneration; *PPV* pars plana vitrectomy; *RPE* retinal pigment epithelium; *SMD* Stargardt’s macular dystrophy; *VA* visual acuity

The largest of these trials evaluated hUTC in a cryopreserved formulation in 35 patients (NCT01226628) [29]. This was followed by a study evaluating treatment in an additional 21 patients (NCT02659098) [7, 30]. In the first study, a scleral cutdown was used to access the subretinal space. This technique was modified to include endoscopy after 30% of the first 10 patients experiences retinal detachments. This adjustment reduced the rate of RDs to 8.7%, though 44% of participants still experience retinal perforations [5]. In the second study, a suprachoroidal approach to subretinal delivery proved safer and effective. There were no retinal perforations or retinal detachments, though subretinal transplantation was not accomplished in three of 21 (14%) subjects [7]. Importantly, no systemic immune modulation was used in either study, and no significant immune reaction or teratoma formation was noted in any participant [5, 7]. Though designed to assess safety, the first study reported 10 patients experienced a more than 10 letter gain and seven patients experienced a greater than 15 letter gain [5]. In contrast, the phase IIb study reported a mean loss of 5.7 letters and no subjects experienced a greater than 15 letter gain. Because of these visual acuity outcomes, a planned randomized, double-masked treatment phase was terminated [7].

Another early phase trial has evaluated the safety and efficacy of hESC-RPE cells (NCT01344993) [31]. This study evaluated increasing doses of cells (50,000 to 150,000) in nine patients with NNV-AMD. After PPV, a suspension of RPE cells was injected subretinally near the border of geographic atrophy. Patients were treated with systemic tacrolimus and mycophenolate mofetil (MMF), but two patients stopped the systemic immunomodulators due to side effects or adverse events. No signs of acute transplant rejection were noted. Subretinal pigmentation postulated to be RPE was observed and increased in size over time for a majority of subjects. Though the study was not powered nor controlled to detect visual acuity effects, four eyes experienced more than 15 letter gains and no eyes lost vision during the follow-up period [21].

A similar implantation technique was used in a small study evaluating 2 patients with advanced NNV-AMD (NCT01674829) [32]. After PPV, the hESCs-RPE cells were injected subretinally. The patients were treated with systemic tacrolimus and MMF. Increasing subretinal pigmentation to varying degrees was noted postoperatively. Visual acuity remained stable in one patient (count fingers) and improved in one patient (20/320 to 20/200) at one-year follow-up. The authors noted that the subretinal pigmentation may represent engrafted RPE; however, this finding cannot be differentiated from phagocytized pigment by macrophages without histology [33].

Another phase I/IIa study evaluated hESC-RPE cells cultured on a synthetic parylene substrate to create a polarized monolayer (NCT02590692) [3, 34]. After PPV, the 3.25 × 6.25 mm hESC-RPE monolayer was implanted subretinally using special insertion forceps in four patients with NNV-AMD. One patient met inclusion criteria but did not have the implant placed due to subretinal fibrinoid debris noted during surgery. The patients were immunosuppressed with tacrolimus alone. There were no severe adverse events related to the RPE cells or immunosuppression. One patient had a larger hemorrhage that required intravitreal treatment with bevacizumab. Though only designed to investigate safety and tolerability, one subject experienced a 17 letter gain. The authors postulated that the beneficial effects on visual acuity were related to the integration of the transplanted RPE with remaining, previously dormant photoreceptors. OCT imaging showed reconstitution of hyperreflective outer retinal bands, likely representing the external limiting membrane and ellipsoid zone [3].

#### Neovascular AMD (NV-AMD)

Two early phase studies have evaluated hESC-RPE therapy for anti-vascular endothelial growth factor (VEGF) recalcitrant NV-AMD patients (Table 2). In a phase I trial, three patients underwent PPV, removal of the subretinal neovascular membrane, and subretinal injection of suspended hESC-RPE cells (NCT02749734) [34, 35]. Patients were immunosuppressed with MMF, prednisone, and tacrolimus. There was no evidence of tumor formation or immune rejection in any patient. At 12-month follow-up, vision improved from HM to 20/400 in 2 patients and remained stable in the third. Subretinal pigmentation was not noted, but a reflective layer developed on OCT imaging and the overlying neural retina thickened. The authors conclude that this suggests that the injected cells may have a positive effect on the neural retina [36].

**Table 2:** Neovascular age-related macular degeneration trials

Trial	Disease	Sample size	VA inclusion criteria	Cell derivation and preparation	Transplantation approach	Immuno-suppression	VA outcomes	Adverse events (AE)
RPE Sheet Case Report UMIN000011929	NV-AMD	2	≤ 20/200	iPSC-RPE sheet	PPV and subretinal delivery	None	Stable vision in 1/2	Cystoid macular edema in 1/2 Transplantation aborted because of concerns about genetic changes that occurred in the iPSCs and iPSC-derived RPE cells in 1/2
HLA-matched iPSC-RPE UMIN000026003	NV-AMD	5	≤ 20/200	iPSC-RPE suspension from HLA homozygous donor	PPV and subretinal delivery	None	> 10 letter gain in 2/5 Stable vision in 3/5	No severe AEs Mild inflammation in 2/5 Epiretinal membrane in 1/5 Aseptic endophthalmitis in 1/5
Q-CTS-hESC-2 NCT02749734	NV-AMD	3	≤ 20/400	hESC-RPE suspension	PPV and subretinal delivery	Tacrolimus, MMF, Prednisone	Improved VA in 2/3 Stable vision in 1/3	No severe AEs
SHEF-1.3 hESC patch NCT01691261	NV-AMD	2	≤ 20/120	hESC-RPE monolayer	PPV and subretinal delivery	Prednisone, Fluocinolone implant	> 20 letter gain in 2/2	Retinal detachment with proliferative vitreoretinopathy in 1/2 Exposure of fluocinolone implant suture in 1/2

*h-ESC* human embryonic stem cells; *HLA* human leukocyte antigen; *iPSCs* induced pluripotent stem cells; *MMF* mycophenolate mofetil; *NV-AMD* neovascular age-related macular degeneration; *PPV* pars plana vitrectomy; *RPE* retinal pigment epithelium; *SMD* Stargardt’s macular dystrophy; *VA* visual acuity

Another small study investigated a “patch” of RPE cells transplanted in NV-AMD subjects (NCT01691261) [37]. hESCs-RPE cells were cultured on a synthetic membrane into a monolayer that was then placed subretinally after PPV. Immunomodulation included oral prednisone and a fluocinolone intraocular implant. Over 12 months, adverse events included a retinal detachment with proliferative vitreoretinopathy and exposure of a fluocinolone implant suture requiring revision. Visual acuity improved by 29 and 21 letters, reading speed improved in by 81 and 48 words/minute, and microperimetry showed fixation over the patch. However, full field ERG did demonstrate reduced photoreceptor function. The authors concluded that this phase I study demonstrated the safety of the patch and sufficiency of local immunosuppression [4].

Two other reports have utilized iPSC to generate RPE cells for treatment of NV-AMD. A 2017 study reported the first implantation of an autologous iPSC-RPE cell layer without a scaffold (UMIN000011929). In two patients, iPSCs were first induced from skin tissue and then differentiated into a sheet of RPE cells. Testing detected aberrant genetic deletions in the RPE cells for one patient, leading the researchers not to implant this RPE sheet. The second patient underwent subretinal placement of the autologous iPSC-RPE sheet without immune suppression. Over 25 months, no graft rejection and no excess proliferation were noted. OCT imaging revealed a hyperreflective RPE-like line extending beyond the initial margin and one area demonstrated possible recovery of photoreceptor cells. Visual acuity remained stable at one-year follow-up [38].

Another trial enrolled five patients with NV-AMD or polypoidal choroidal vasculopathy (UMIN000026003). The iPSCs were harvested from a homozygous HLA matched donor. After PPV, a suspension of iPSCs-RPE cells was injected subretinally. Only subtenon’s triamcinolone was used for immunosuppression. After 52 weeks of follow-up, no adverse events were observed. All patients developed epiretinal membranes, and one required surgical membrane peel. Analysis showed that this membrane contained pigmented cells positive for RPE markers. One case of sterile endophthalmitis secondary to triamcinolone was noted. All subjects had increased subretinal pigmentation, but the location of pigment was not substantially in the macula in 3/5 cases, likely related to suboptimal injection location or technique. The authors also noted that backflow of graft cells into the vitreous was an issue that needs to be investigated further [24].

### Stargardt's macular dystrophy (SMD)

Two studies discussed above included both SMD and AMD patients (NCT01674829 and NCT01345006) [32, 39]. The SMD cohorts included two and nine participants, and all were treated with subretinal injection of hESC-RPE suspensions (Table 3) [19, 31]. In the smaller study, one patient did not develop pigmentation after transplantation, but vision improved by 13 letters. Increased subretinal pigmentation was noted in the second subject, and vision improved by 19 letters. Of note, the vision in the fellow eye improved by nine letters without intervention in both patients. This study was designed to assess safety, and the authors did not identify any significant concerns [33].

**Table 3:** Stargardt’s macular dystrophy clinical trials

Trial	Disease	Sample size	VA inclusion criteria	Cell derivation and preparation	Transplantation approach	Immuno-suppression	VA outcomes	Adverse events (AE)
South Korean Phase 1/2a NCT01674829	SMD/ NNV-AMD	2 SMD	≤ 20/320	hESC-RPE suspension	PPV and subretinal delivery	Tacrolimus and MMF	Improved VA in 2/2	Herpetic vesicles on skin in 1/2
MA09-hRPE cell line Phase 1/2a NCT01469832	SMD	12 (3 dose escalation cohorts)	≤ 20/400	hESC-RPE suspension	PPV and subretinal delivery	Tacrolimus and MMF	Stable vision in 12/12	Retinal tears and dialysis in 1 /15 Subretinal hemorrhage in 2/15 Vitreous hemorrhage in 1/15 Immunosuppression side effects (HSV, lethargy, headache, nausea) in 5/15
Advanced Cell Technology Phase 1/2a NCT01345006	SMD/ NNV-AMD	9 SMD (3 dose escalation cohorts)	≤ 20/200	hESC-RPE suspension	PPV and subretinal delivery	Tacrolimus and MMF	> 15 letter improvement in 3/9 Stable vision in 3/9 > 10 letter decrease in 1/9	Preretinal pigmentation in 1/9 Endophthalmitis 1/9 Vitreous inflammation with vitreous band formation in 1/9

*h-ESC* human embryonic stem cells; *HSV* herpes simplex virus; *MMF* mycophenolate mofetil; *NNV-AMD* non-neovascular age-related macular degeneration; *PPV* pars plana vitrectomy; *RPE* retinal pigment epithelium; *SMD* Stargardt’s macular dystrophy; *VA* visual acuity

The larger study divided participants into three dose escalation cohorts. A suspension of hESC-RPE cells was injected subretinally near atrophy. Patients were treated with systemic tacrolimus and MMF. One patient developed *S. epidermidis* endophthalmitis four days after surgery, which required antibiotic treatment and cessation of immunosuppression. No signs of immune rejection were noted, and the authors noted that the younger SMD patients tolerated immune suppression better than older AMD subjects. Subretinal pigmentation postulated to be RPE was noted at the border of retinal atrophy and increased in size over time for a majority of subjects. Though not designed to detect visual acuity effects, three eyes experienced more than 15 letter gains and one eye lost more than 10 letters over 12 months of follow-up [21].

The largest study evaluating the safety of hESC-RPE in SMD included 12 patients (NCT01469832) [40]. In this dose escalation study, a suspension of 50,000 to 200,000 hESC-RPE cells was transplanted subretinally after PPV. Subjects were treated with tacrolimus and MMF without complication. Surgical complications in four patients included retinal dialysis, subretinal hemorrhage, and vitreous hemorrhage. There was no evidence of immune rejection or adverse proliferation of RPE cells. Subretinal pigmentation was observed in all participants, and OCT imaging showed a hyperreflective layer consistent with RPE. The authors reported a correlation between the dose of injected cells and the area of pigmentation, though the highest dose did result in overlying retinal thinning. There was no significant improvement or decline in VA, ERG testing, or microperimetry sensitivity in the 12 participants, possibly related to the advanced disease in all patients [22].

### “Cell therapy” clinics

As detailed above, legitimate studies have enrolled a small number of RDD patients to date and are primarily focused on evaluating the safety of cell therapies. There are no FDA approved cell-based therapies for retinal conditions at this time. However, “cell therapy” clinics exist and provide non-FDA approved, unproven treatments without proper regulatory oversight. These clinics targeted patients through social medial and direct-to-consumer advertising and charge patients high out-of-pocket fees for these treatments. It can be difficult to differentiate these therapies from legitimate clinical trials [41]. Despite efforts to educate the public and highlight the fact that these treatments are not FDA approved, these clinics remain an ongoing problem.

Serious ocular complications after intravitreal injection of stem-cells in these clinics have been repeatedly observed. In one report, after paying $5000 to undergo treatment with adipose tissue-derived stem cells, five of six eyes experienced blinding complications, including retinal detachments. Further reports described retinal detachments after intravitreal injections of adipose-derived stem cells in other unregulated clinics [42, 43]. Similarly, subretinal injection of autologous bone marrow-derived stem cells at a “cell therapy” clinic in a patient with a history of SMD was complicated by [44].

Despite the publicity of these complications and recent increased FDA oversight, these treatments continue to be available. A study by Nirwan et al. reported that 40 companies advertised “cell therapies” for ocular diseases in 76 clinics in the USA. Patients are charged between $4,000 to $10,500 to undergo treatment. Interventions utilize a range of cell sources, primarily autologous adipose tissue (67%). Patients with macular degeneration are commonly targeted by these clinics, though treatments for optic neuritis, retinitis pigmentosa, and diabetic retinopathy are also advertised [45]. This study emphasizes that there are still a high number of clinics that offer direct-to-consumer cell therapy. Taken together, these reports highlight the need for further regulation of these clinics and patient education about the potential complications from unproven treatments.

## Conclusion

Currently, there are no FDA-approved cell-based treatments for RDDs, though there are several trials investigating potential therapies. Though not powered to demonstrate efficacy, legitimate clinical trials have reported favorable safety profiles in NNV-AMD, NV-AMD, and SMD. These early phase studies have shown that multiple surgical approaches with cells derived from hESCs, iPSCs, and hUTDs are feasible. However, the necessary data demonstrating the efficacy and long-term safety of these treatments are still pending. Nevertheless, “cell therapy” clinics continue to conduct direct-to consumer marketing for non-FDA-approved treatments for ocular conditions with potentially blinding complications.

## Data Availability

Not applicable.

## Abbreviations

ERG: Electroretinogram
FDA: Food and Drug Administration
hESCs: Human embryonic stem cells
HLA: Human leukocyte antigen
hUTDs: Human umbilical tissue-derived cells
iPSCs: Induced pluripotent stem cells
MMF: Mycophenolate mofetil
NNV-AMD: Non-neovascular age-related macular degeneration
NV-AMD: Neovascular age-related macular degeneration
OCT: Optical coherence tomography
PPV: Pars plana vitrectomy
RDD: Retinal degenerative disease
RP: Retinitis pigmentosa
RPE: Retinal pigment epithelium
SMD: Stargardt’s macular dystrophy
